# Biological Evaluation of Isoniazid Derivatives as an Anticancer Class

**DOI:** 10.3797/scipharm.1307-25

**Published:** 2013-09-22

**Authors:** Felipe A. R. Rodrigues, Augusto C. A. Oliveira, Bruno C. Cavalcanti, Claudia Pessoa, Alessandra C. Pinheiro, Marcus V. N. de Souza

**Affiliations:** 1 Laboratório de Oncologia Experimental, Universidade Federal do Ceará, Fortaleza, CE, Brazil.; 2 FioCruz-Fundação Oswaldo Cruz, Instituto de Tecnologia em Fármacos-Far-Manguinhos, Rua Sizenando Nabuco, 100, Manguinhos, 21041-250 Rio de Janeiro, RJ, Brazil.

**Keywords:** Antitumor activity, Isoniazid, Hydrazone, Drugs, Cytotoxicity

## Abstract

A series of thirty-two isoniazid derivatives have been evaluated for their activity against four human cancer cell lines with potent cytotoxicity (IC50 ranging from 0.61 to 3.36 μg/mL). The structure-activity relationship (SAR) analysis indicated the number, the positions, and the types of substituents attached to the aromatic ring as being critical factors for the biological activity. Briefly, we observed that the presence of a hydroxyl group on the benzene ring plays an important role in the anticancer activity of this series, especially when it is located in *ortho*-position. Among the thirty-two compounds, three displayed good cytotoxic activity when compared to the reference drug doxorubicin and are thus being considered leading compounds of this new class.

## Introduction

**Fig. 1:**
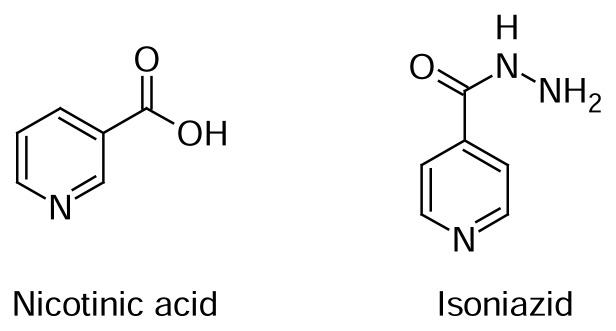
Structures of Nicotinic acid and Isoniazid

Nicotinic acid (pyridine-3-carboxylic acid), its derivatives and isomers form an important class of heterocyclic compounds with a wide range of applications, among which the use thereof as starting materials for the synthesis of biological active compounds such as Nevirapine, namely an anti-HIV drug [1]. Nicotinic acid, also known as vitamin B3 and niacin, as well as its amide niacinamide are found in several aliments and animals, and play a critical role in different biological processes [2]. This class of heterocyclic compounds also showed a broad spectrum of biological activities, such as anti-carcinogenic [3], antioxidant [4], anti-inflammatory [5], and anti-bacterial ones [6]. For instance, we should mention isoniazid (isonicotinylhydrazine), an important first-line anti-tuberculosis drug, which keeps an analogy with isonicotinic acid, an isomer of nicotinic acid (Figure 1) [7, 8]. However, in spite of the relevance of isoniazid in tuberculosis treatment in the last twenty years, this drug has rarely been studied in the field of cancer [9], even after promising perspectives of analogues of isoniazid in this field, which can be illustrated by the work of Malhotra and co-workers [10]. This disease, which according to an estimate from the National Institute of Health (NIH), implied in overall costs of $226.8 billion in 2007 [11], accounted for 7.6 million deaths (13% of all deaths) in 2008 [12], being to date a leading cause of death worldwide. Therefore, in view of such an urgent need of new drugs against this disease, an important strategy for drug discovery has been recently developed, namely drug repositioning, which is defined as the study aimed at the application of available drugs for other diseases [13, 14]. This study of isoniazid and its derivatives in the cancer field, which shall be grounded in our experience with TB drugs [15–20], is particularly motivated by the lack of studies in drug discovery focused on isoniazid derivatives against cancer. In this context, the aim of this work was the antitumoral evaluation against human cancer cell lines of thirty-two isoniazid hydrazone derivatives designed by molecular hybridization (Scheme 1), which display potent and promising results (Tables 1 and 2). Hydrazones are also described to possess a wide range of pharmacological activities, such as being anticancer agents [21, 22].

## Results and Discussion

### Chemistry

All the isonicotinohydrazides derivatives **1**–**32** were synthesized by our research group and tested against *M. tuberculosis* [15–18]. Briefly, the synthesis of desired compounds involved the reaction of appropriate benzaldehydes and isoniazid, in THF under reflux or room temperature for 4–12 hours. The compounds were obtained in 75–99% yields.

### Cytotoxicity Against Cancer Cell Lines

All compounds **1–32** were tested *in vitro* against three human cancer cells: OVCAR-8 (ovary), SF-295 (glioblastoma), and HCT-116 (colon) (National Cancer Institute, Bethesda, MD) at 5 μg/mL by using the MTT assay (Table 1). Afterward, the compounds were classified by their growth inhibition (GI) percentage, at least in one cell line, as active (100% GI), moderately active (75% < GI < 100%), or inactive (GI < 50%).

Compounds **15**, **18,** and **31**, which displayed more than 96% of GI, were selected for *in vitro* anticancer activities evaluation against four human cancer cell lines: HCT-116 (colon), OVCAR-8 (human ovary), HL-60 (leukemia), and SF-295 (glioblastoma), using the MTT assay. The concentrations that induce 50% inhibition of cell growth (IC50) in μg/mL are reported in Table 2.

**Fig. 2:**
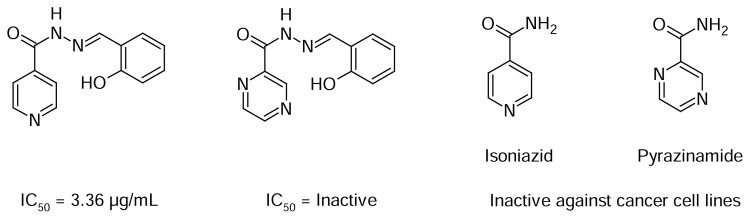
Pyrazinamide and related compounds

The structure-activity relationship (SAR) analysis indicated that the number, the positions, and the types of substituents attached to the aromatic ring are critical for the biological activity. The disubstituted derivatives displayed the best results appearing as the most active groups attached to the ring, namely the hydroxy, methoxy, chloro, and nitro groups. In general, we observed that the presence of hydroxyl groups on the benzene ring plays an important role in the anticancer activity of this series, especially when it is located in *ortho-*position. It is worth mentioning that, given that hydroxyl groups located in *ortho-*position in hydrazone systems are good ligands for metals, the action mechanism of this class could possibly be based on the formation of complexes that are likely to inactivate enzymes involved in abnormal cell division. As another peculiarity about the SAR from this class, we should mention that when comparing one of the leads from this class to the bioisostere (*E*)-*N*′-(2-hydroxybenzylidene)pyrazine-2-carbohydrazide [20, 23], this lead displayed a stronger antitumor activity, thus indicating that the inclusion of another nitrogen into the ring and/or the position of the group acylhydrazone decreases the potency of the pyridine hydrazone class. This bioisostere is an analogue of the first-line drug pyrazinamide, which is also an important first-line anti-tuberculosis drug (Figure 2) [8].

## Experimental

### General Procedure for the Synthesis of Isoniazid Hydrazone Derivatives 1–32

To a stirred solution of isoniazid (1.0 mmol) in ethanol (10 mL) was added the appropriate amount of benzaldehyde (1.05 mmol), and the reaction mixture was stirred for 4–12 hours at room temperature or under reflux. The reaction mixture was concentrated under reduced pressure, and the residue was purified by washing with cold ethanol (3 × 10 mL), thus affording the isoniazid hydrazone derivatives **1–32** in 75–99% yield.

### Cytotoxicity Against Cancer Cell Lines

Compounds **1–32** (1.715–5.0 μg/mL) were tested for their cytotoxic activity against 3–4 human cancer cell lines: OVCAR-8 (ovary), SF-295 (glioblastoma), HCT-116 (colon), and HL-60 (leukemia) (National Cancer Institute, Bethesda, MD). All cell lines were maintained in RPMI 1640 medium supplemented with 10% fetal bovine serum, 2 mM glutamine, 100 U/mL penicillin, and 100 μg/mL streptomycin at 37 °C with 5% CO_2_. Each compound was dissolved with DMSO until reaching a concentration of 1 mg/mL. The final concentration of DMSO in the culture medium was kept constant, below 0.1% (v/v). Compounds **1–32** were incubated with the cells for 72 hours. The negative control received the same amount of DMSO (0.001% in the highest concentration). The cell viability was determined by reduction of the yellow dye 3-(4,5-dimethyl-2-thiazol)-2,5-diphenyl-2*H*-tetrazolium bromide (MTT) to a blue formazan product as described by Mosmann [24].

## Conclusion

In this work, we report the potent cytotoxic activity of a series of thirty-two isoniazid hydrazone derivatives, which have been evaluated for their activity against four human cancer cell lines. The SAR of this class indicated that the number, the positions, and the types of substituents attached to the aromatic ring are critical for the biological activity. Another peculiarity about the SAR from this class concerns the fact that bioisostere (*E*)-*N*′-(2-hydroxybenzylidene)pyrazine-2-carbohydrazide displayed a lower antitumor activity, which indicates that the inclusion of another nitrogen into the ring and/or the position of the group acylhydrazone decreases the potency of the pyridine hydrazone class.

In comparison to the reference drug doxorubicin, compound **18** displayed a good cytotoxic activity, thus suggesting that compounds based on the isoniazid drug could be a good starting point for the discovery of new leading compounds against cancer.

## Figures and Tables

**Sch. 1:**
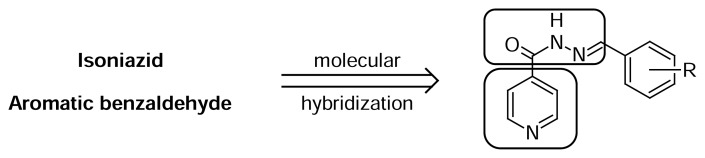
Isoniazid hydrazone derivatives designed by molecular hybridization

**Tab. 1:** Growth Inhibition Percentage (GI %) for three Tumors Cell Lines by the MTT Assay of compounds **1–32**.

Cpd.	R	Growth Inhibition^a^ (%)					
		
		OVCAR-8	SD	SF-295	SD	HCT-116	SD
1	H	0.00%	0.00%	19.31%	1.54%	0.00%	0.00%
2	2-NO_2_	0.74%	1.40%	26.54%	3.79%	9.31%	6.01%
3	3-NO_2_	2.00%	0.89%	29.81%	1.81%	0.00%	0.00%
4	4-NO_2_	0.00%	0.00%	28.76%	0.82%	0.00%	0.00%
5	2-F	0.00%	0.00%	23.04%	0.00%	0.00%	0.00%
6	3-F	0.00%	0.00%	34.53%	0.25%	8.04%	1.17%
7	4-F	0.00%	0.00%	24.38%	4.70%	1.70%	6.19%
8	2-Cl	0.00%	0.00%	32.20%	8.00%	0.00%	0.00%
9	3-Cl	0.00%	0.00%	34.41%	4.70%	2.78%	3.23%
10	4-Cl	0.00%	0.00%	28.47%	7.34%	5.00%	0.63%
11	2-Br	62.18%	5.97%	65.02%	1.20%	66.87%	4.13%
12	3-Br	0.00%	0.00%	34.41%	3.71%	6.27%	1.53%
13	3-CN	0.00%	0.00%	40.13%	2.56%	17.94%	1.50%
14	4-CN	0.00%	0.00%	31.38%	0.41%	4.74%	0.63%
**15**	**2-OH**	**100.00%**	**1.78%**	**80.47%**	**3.38%**	**80.58%**	**0.54%**
16	3-OH	0.00%	0.00%	22.34%	3.46%	0.00%	0.00%
17	4-OH	0.00%	0.00%	7.36%	6.68%	0.00%	0.00%
**18**	**2,3-diOH**	**100.00%**	**0.13%**	**86.53%**	**1.90%**	**79.44%**	**0.36%**
19	3,4-diOH	36.31%	1.65%	37.39%	0.33%	26.07%	4.04%
20	2-OCH_3_	2.27%	5.84%	43.80%	0.33%	0.00%	0.00%
21	3-OCH_3_	1.19%	1.27%	22.34%	1.81%	3.66%	0.90%
22	4-OCH_3_	5.95%	0.02%	23.45%	4.04%	0.00%	0.00%
23	2,3-diOCH_3_	0.65%	1.78%	26.95%	0.25%	0.00%	0.00%
24	2,4-diOCH_3_	19.87%	2.29%	43.74%	6.18%	0.00%	0.00%
25	2,5-OCH_3_	49.52%	1.91%	52.78%	6.43%	66.05%	1.17%
26	2,6-diOCH_3_	23.65%	1.67%	35.17%	5.94%	72.46%	2.51%
27	3,5-diOCH_3_	7.48%	0.25%	39.13%	5.11%	15.85%	1.23%
28	2,3,4-triOCH_3_	0.00%	0.00%	32.90%	1.57%	0.00%	0.00%
29	2-OCH_2_CH_3_	2.63%	0.51%	37.91%	0.25%	0.00%	0.00%
30	3-OCH_2_CH_3_	0.00%	0.00%	9.69%	4.70%	0.00%	0.00%
**31**	**2-OH; 3-OCH_3_**	**100.00%**	**1.27%**	**86.77%**	**0.08%**	**88.64%**	**0.27%**
32	3-OH; 4-OCH_3_	18.89%	6.48%	43.86%	1.79%	5.76%	1.48%

a Experiments were performed in triplicate.

SD … Standard Deviation.

**Tab. 2:** Cytotoxic activity of compounds **18**, **31**, and **15** [IC_50_ (μg/mL)] on tumor cell lines*.

Cpd.	HCT-116 IC_50_ SD	OVCAR-8 IC_50_ SD	HL-60 IC_50_ SD	SF-295 IC_50_ SD
15	2.025 1.427 to 2.873	2.021 1.857 to 2.199	2.452 2.174 to 2.766	3.366 1.814 to 6.245
18	1.367 1.106 to 1.690	0.6182 0.5522 to 0.6922	0.6173 0.5421 to 0.7028	0.9670 0.8281 to 1.129
31	1.718 1.133 to 2.606	1.242 1.059 to 1.455	1.932 1.646 to 2.268	1.912 1.621 to 2.256
Doxorubicin	0.125 (0.09–0.17)	0.265 (0.17–0.305)	0.02 0.01–0.02	0.23 0.19–0.25

* Data are presented as IC_50_ values and 95% confidence intervals obtained by nonlinear regression for all cell lines colon (HCT-116), ovarium (OVCAR-8), (leukemia (HL-60), glioblastoma (SF-295), from three independent experiments. Doxorubicin (Dox) was used as positive control. Experiments were performed in triplicate. IC_50_ = concentrations that induce 50% inhibition of cell growth in μg/mL.
